# An Evaluation of Symmetry in the Lower Limb Joints During the Able-Bodied Gait of Women and Men

**DOI:** 10.2478/v10078-012-0078-5

**Published:** 2012-12-30

**Authors:** Wanda Forczek, Robert Staszkiewicz

**Affiliations:** 1 Department of Biomechanics, University School of Physical Education, Krakow, Poland.

**Keywords:** angular changes, relative asymmetry index, bilateral data, Vicon

## Abstract

For many years, mainly to simplify data analysis, scientists assumed that during a gait, the lower limbs moved symmetrically. However, even a cursory survey of the more recent literature reveals that the human walk is symmetrical only in some aspects. That is why the presence of asymmetry should be considered in all studies of locomotion. The gait data were collected using the 3D motion analysis system Vicon. The inclusion criteria allowed the researchers to analyze a very homogenous group, which consisted of 54 subjects (27 women and 27 men). Every selected participant moved at a similar velocity: approximately 1,55 m/s. The analysis included kinematic parameters defining spatio-temporal structure of locomotion, as well as angular changes of the main joints of the lower extremities (ankle, knee and hip) in the sagittal plane. The values of those variables were calculated separately for the left and for the right leg in women and men. This approach allowed us to determine the size of the differences, and was the basis for assessing gait asymmetry using a relative asymmetry index, which was constructed by the authors. Analysis of the results demonstrates no differences in the temporal and phasic variables of movements of the right and left lower limb. However, different profiles of angular changes in the sagittal plane were observed, measured bilaterally for the ankle joint.

## Introduction

Bipedal gait is essentially a human activity and is only apparently an easy task. Visual inspection of the gait in healthy people seems to indicate that this way of locomotion should be smooth, symmetrical, and carried out with steps of equal length. In addition, such an analysis assumed a similar strategy of movement for the right and left leg. This arrangement of transferring the body in humans was described by many authors, including Vaughan et al. (1992). Such scientific judgments were not isolated, and many subsequent researchers consequently assumed that the lower limbs behave in a symmetrical manner during gait (Eng and Winter, 1995; Hannah et al., 1984). However, the procedure followed was not correct since it was based on a lack of a satisfactory definition of symmetry in gait; symmetry was defined as perfect agreement between external kinematics of the left and right limbs (Herzog et al., 1989). Meanwhile, asymmetry was regarded as the lack of a statistical difference for parameters measured bilaterally (Gabbard, 1997). Therefore, asymmetry was sometimes considered to indicate gait pathology (De Stefano et al., 2004). At the same time, studies such as Bishop et al. (2002) and Grouios (2005) found evidence of asymmetry in normal ambulation, leading them to characterise it as a natural state, inseparably associated with locomotion.

A review of the literature indicates that the human gait is symmetrical, but only in certain respects. That is why the presence of asymmetry should be taken into account in all studies including analyses of the lower limb movements (Macellari et al., 1999; Maupas, 2002). This aspect, however, is still underestimated. Different outcomes in this area are mainly due to one of three factors: methodological assumptions that lead to simplifications in the data interpretation (Hamill et al., 1984; Ounpuu et al., 1991), the small number of subjects in the study (Dickey and Winter, 1992; Crowe et al., 1993), or, finally, the conclusions are based on unilateral data collection (Sutherland et al., 1980; Yang et al., 1985).

It is worth noting that so far there has been some important research which documents gait asymmetry in kinematics (Allard et al., 1996; Shorter, 2008), spatio-temporal parameters (Macellari et al., 1999; Sadeghi, 2003), kinetics (Herzog et al., 1989; Seeley et al., 2008) and electromyographic activity (Arsenault et al., 1986; Gundersen, 1989). Those providing different methods to identify asymmetry with symmetry indices and ratios are particularly noteworthy (Robinson et al., 1987; Herzog et al., 1989; Vagenas and Hoshizaki, 1992; Shorter et al., 2008). The parameters used in the indices have included plantar pressure distribution, vertical component of ground reaction forces, speed and stride frequencies (Sadeghi, 2003). However, such assessment does not provide information on the behavior of the joints throughout the gait cycle.

### Aim of the study

Analysis of the literature encouraged the authors to raise the problem of gait asymmetry and verify the conflicting reports. Because of the aforementioned contradictions, we developed our own approach to the explanation of the unequal patterns of motion between the left and right lower limb. The primary focus of this study was to develop an insight into kinematic asymmetry in gait at natural speed in both women and men. For this reason, we utilized a method which took into account the precision of the acquisition system.

## Material and Methods

The registration of natural preferred-speed gait was carried out in the Department of Biomechanics at the University School of Physical Education in Krakow using the three-dimensional motion-analysis system *Vicon* and *Golem* model (Oxford Metrics Ltd.; Oxford, UK). The study population comprised 78 healthy subjects (35 women, 43 men), aged between 18–25 years, recruited from the students of different faculties of two universities: University School of Physical Education in Krakow and the Jagiellonian University. None of them had practiced sport competitively. The morphological characteristics of the group are described in Table 1.

The subjects had no previous history of orthopedic ailment such as injury or surgery, which could affect their walking pattern. According to Kaufman et al. (1996), asymmetrical gait is not observed in able-bodied subjects unless the length discrepancy reaches at least 2 cm. We decided to take 1 cm as our cut-off level: individuals having limb length discrepancies of 1 cm or more were excluded from the study. To meet the recommendation of the Golem model, the length of a subject’s leg was estimated using the distance between the anterior superior iliac spine and lateral malleolus, measured when the subject was standing in a static position.

The inclusion criteria aimed at obtaining a very homogeneous group in terms of walking speed. To ensure consistency, after data collection, we selected only those subjects whose speed was closest to 1.55 m/s, since this value is estimated as close to natural in the able-bodied adult population (Nymark et al., 2005). The preferred walking speed for each subject was determined from the mean speed of 8 walking trials. This requirement was met by only 54 subjects: 24 subjects whose speed was different from the set values of 1.55 m/s were excluded. So finally, the measurements were carried out on a sample of 27 women and 27 men.

For each of the subjects we registered 20 gait cycles (40 steps). After hearing the signal the subject covered a distance of about 50 meters. From the collected data we were able to identify kinematic variables describing the temporal and phasic structure of locomotion, as well as the angular changes in the major joints of the lower limbs (ankle, knee and hip) in the sagittal plane. The values of these parameters were calculated separately for the left and right leg, which made it possible to determine the size of the differences and was the basis for assessing gait asymmetry. Body segments were defined by means of 39 reflective markers having a diameters of 25 mm attached to the head, trunk, pelvis, arms and legs.

Kinematic data were divided into individual gait cycles for each side of the body. A gait cycle was defined from heel strike to subsequent heel strike. Data for each cycle were normalized (0% GC – 100% GC). For the purpose of analysis, the functional phases of gait were subdivided into (according to Perry, 1992) LR-loading response (10% GC), MST-mid stance (20% GC), TST-terminal stance (20% GC), PSW-pre swing (10% GC), ISW-initial swing (10% GC), MSW-mid swing (15% GC), and TSW-terminal swing (15% GC).

To assess the normal distribution of acquired data we used the Shapiro-Wilk test. The student’s t test for independent groups was used to examine the statistical significance of differences between mean values of variables obtained during gait. To determine the average level of diversification of the parameters in terms of gender in the characteristic phases of a standardized gait cycle, which is described below, we applied a two-way analysis of variance *ANOVA* with repeated measurements.

To evaluate the level of gait asymmetry in the angular data, the authors employed a relative asymmetry index (RAI):

(1) $$\text{RAI}=\frac{\bar{\text{X}}}{\text{Y}}100\%,\text{where}:$$

x̄ - the average difference between the values noted for the right and left limbs in a given phase of the gait cycle (LR, MST, etc.)
Y - total range of motion of the angular changes in the given phase (absolute value of the difference between the largest and the smallest angles for a given phase of the gait cycle).

The average difference (*x̄*) in successive phases of gait was calculated according to the following formula:

(2) $$\bar{\text{X}}=\frac{\sum_{\text{i}=\text{l}}^{\text{i}=\text{n}}\left|{\text{R}}_{\text{i}}-{\text{L}}_{\text{i}}\right|}{\%\text{GC}},\text{where}:$$

R, L- instantaneous value of the angle of individual joints in the right and left lower limb, % GC - relative duration of the given phase in the gait cycle (number).

Consistently, in accordance with the adopted symbols and the way of their determination, the described equation for LR phase (10% GC) was as follows:

(3) $${\bar{\text{X}}}_{\text{LR}}=\frac{\sum_{\text{i}=\text{l}}^{\text{i}=10}\left|{\text{R}}_{\text{i}}-{\text{L}}_{\text{i}}\right|}{10}.$$

## Results

Tables 2 and 3 show the values of selected kinematic parameters of gait, both in terms of gender and the side of the body. As you can see (Table 2), walking at a speed of 1.55 m/s was achieved by women with a higher step frequency than by men. This difference was about 5% and was statistically significant. As a consequence of higher frequency of women’s gait, lower values were recorded for almost all variables, the exception being that the double support phase in both groups was almost the same.

The largest relative difference of the values measured in women and men was approximately 5% and occurred during the single support phase (SS). Simultaneously, the smallest difference (3.2%) was recorded for the stride (s). Details of basic statistical analysis are presented in Table 3. Analyzing this table, bearing in mind the aforementioned variables describing movement of the right and left lower limb (t_GC_, s, SS, DS), it should be emphasized that there were no differences recorded for both legs.

**Figure 1:**
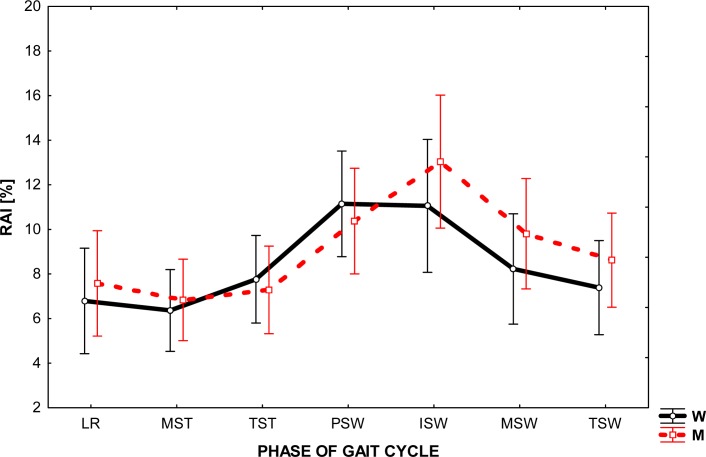
The level of relative asymmetry index (RAI) in the given phase of gait cycle in the ankle joint in women (W) and in men (M)

Figure 1 illustrates the values of the asymmetry index for the ankle movements in the sagittal plane during the gait of men and women in one gait cycle (GC) divided into phases. RAI values ranged from about 6 to 13%, providing a significant difference in the profile of both of these joints. For both sexes, the greatest difference in the values registered for the left and right ankle was the boundary between stance and swing of the limb (PSW, ISW). In this part of the gait cycle RAI value reached 13% in men whereas in women it reached about 10%. In addition, regardless of gender, there were greater values of relative asymmetry index in the swing than stance phase, but such a phenomenon is more noticeable in men.

A comparison of the RAI level for major joints of the lower extremity reveals its highest value was for the ankle joint. This observation was carried out for both men and women in all phases of the gait cycle (0%GC – 100%GC).

**Figure 2:**
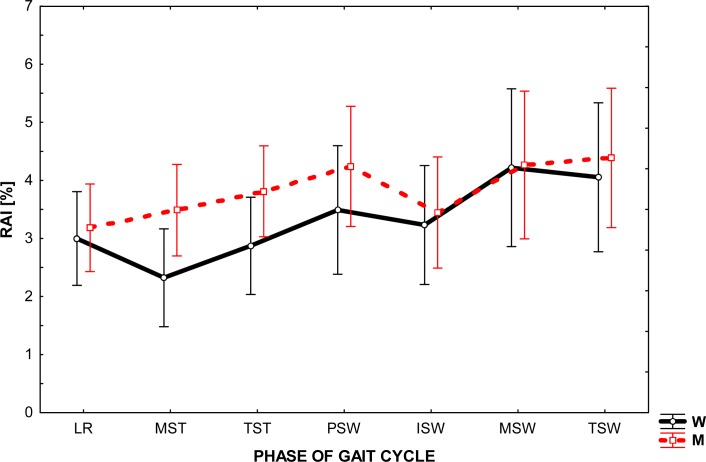
The level of relative asymmetry index(RAI) in the given phase of gait cycle in the knee joint in women(W) and in men (M)

As shown in Figure 2, the mean values of the asymmetry index, which describes the angular changes in the knees in the sagittal plane during ambulation of men and women, were similar and ranged from 2 to 4%. Analyzing the data, one can see that the largest value of that interval was recorded in the phase of swing, while the RAI value variation throughout the cycle was small.

At the same time, the results revealed some differences in the bilateral work of knee joints, which are reflected in the way of locomotion of both sexes. Observation of the RAI shows that from the beginning to the end of the cycle (from LR to TSW), a greater asymmetry is noticed in men. It should be noted, however, that this trend was recorded at a relatively low level of RAI, as mentioned earlier.

**Figure 3:**
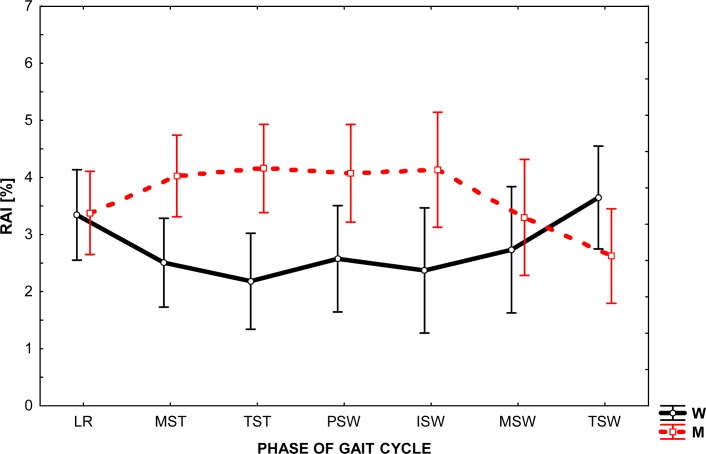
The level of relative asymmetry index (RAI) in the given phase of gait cycle in the hip joint in women (W) and in men (M)

RAI values, which illustrate asymmetry in the behavior of the left and right hip joint in sagittal plane during gait, are shown in Figure 3. In both groups, they remain at the relatively low similar level of 2 to 4% of the difference. At the same time, it is apparent that the maximum values for women were recorded at the beginning and end of the cycle (LR, TSW), while in men in the middle of the cycle (MST, TST, PSW, ISW). The data show that a generally greater asymmetry occurs in the hip motion during walking in men than in women. A detailed analysis of the results provides evidence that not only the character of changes throughout the whole range is different in both groups. The recorded value of RAI shows twice the asymmetry in the movement of men. In particular, it was clearly seen between 20 and 70% GC. It should be emphasized that all these phenomena were recorded at low values of RAI and within their narrow range.

## Discussion

The literature is often contradictory with regard to gait asymmetry. There are numerous studies which document gait asymmetry as a consequence of certain pathologies within the human motor system (Finestone et al., 1991; De Stefano et al., 2004). Others have reported unequal patterns of motion between the left and right side as natural phenomenon inextricably associated with bipedal locomotion (Sadeghi et al., 2000; Duhaime, 2000; Bishop et al., 2002; Grouios, 2005). Perhaps this scientific dispute would be resolved more easily if not for the fact that these teams of researchers based their conclusions only on selected gait parameters measured on quite a small sample of subjects.

As we know, the basic parameters of gait are speed and frequency. As a consequence of changes within each of them, there is a change in other derivatives of the spatio-temporal parameters (Bober, 1985; Riley et al., 2001). Analysis of our own results has revealed that kinematic variables measured bilaterally in terms of time did not differ (Table 1). Some data in the available literature contrasts with these findings. Sutherland et al. (1980), Law (1987), Gundersen et al. (1989), Allard et al. (1996) and Macellari et al. (1999) noted differences in kinematic parameters between the right and the left extremities in normal gait. These discrepancies are likely to be a result of differences in gait speed. In the present study we investigated natural gait at a speed of approximately 5.4 km/h. This value is regarded as typical for the able-bodied adult population. In the previous studies, the authors used a wider range of speeds, including trials at much lower speeds. This observation is important in face of the existing results (Staszkiewicz et al., 1999) which indicate that differences of kinematic gait parameters registered for the right and left sides of the body increases when the speed of movement decreases. It seems that the conclusions of all studies could be standardized if comparisons referred to a similar speed of movement.

Analysis of the graphs presented in Figures 1–3 shows that the angular changes in the sagittal plane measured bilaterally are different. The values of RAI calculated on the basis of these differences are greatest for ankle (from 6 to 13%) and are much lower (2 – 4%) for other joints.

The main difficulty arising directly from the obtained asymmetry index values was their interpretation. Our aim was to answer the question: What level of RAI can be recognized as the occurrence of asymmetry in the joint behavior? Because the authors’ method of research differed from that employed by other researcher in the field, the present research results find little confirmation in the available literature. We therefore considered the level of 5% of RAI as the boundary between the phenomena of symmetry / asymmetry in gait, assuming that a value greater than 5% shall indicate asymmetry. Consequently, the results seem to establish a significant asymmetry between the working of the ankle joint of the left and right lower extremity during natural gait and very slight asymmetry in the knee and hip joints movement.

Given the functional connection between the joints within the lower limb, the interpretation of the results provides some difficulties. During gait, ankle, knee, and hip angles undergo various changes to stabilize the upper body and to provide continual progression. It is natural that any changes in the ankle joint influence the knee and hip as the limb is a kinematic chain. One of the most spectacular forms of cooperation of these parts of the musculoskeletal system is a functional shortening of the lower limb during gait. Optimal performance of this task in the swing phase is possible only when the ankle plantar flexion occurs together with the knee and hip flexion. So it seems that greater asymmetry in the ankle than in the knee and hip movement may be a consequence of their location on the distal end of the aforementioned kinematic chain.

A more likely explanation for the asymmetry in the mobility of ankle joints can be based on two phenomena, which have been described in the literature and are fully confirmed by the results of our own research. First of all, Duhaime (2000) and Sadeghi et al. (2001) give the evidence that the terminal stance and initial swing phase showed the highest dynamics of change. And secondly, at the time of foot off, the last element in the kinematic chain, which is the foot, becomes free, which provides a more specific pattern of motion (Allard et al., 1997). As can be seen, the cited statements are closely connected with the technique of movement, and that is particularly individualized (Norkin and Levangie, 1992). The impact of individual gait pattern and, consequently, the way of placing the feet on the ground, manifests itself even in the degree of wear of soles of the left and right shoes.

From a technical point of view, the study of changes in the knee and hip angle is much easier than in the ankle. It is easier to find publications on this subject, but more detailed reports on asymmetry phenomenon in terms of lower limb joints behavior are quite scarce. As mentioned above, the obtained results reveal a very slight angular variation of the left and right knee, which is in line with the general opinion supported by the literature. The thesis of a higher level of symmetry in the sagittal plane is developed in the studies of Sadeghi et al. (2001) or Forczek and Chwała (2005). These researchers have found evidence of significant changes in the dynamics only at the end of the support and early swing. Slight differences in the selected angle values in the knees were also noticed by Gundersen et al. (1989) and Wheelright et al. (1993), the latter author combined these observations with the phenomena of lateralization. In turn, Hannah et al. (1984) emphasized a symmetric profile in the knee behavior in the sagittal plane.

The hip joint, due to the anatomical structure and function that it fulfills in the human motor system, may be treated as an extremely stable construction. This part of the body, as a link between the structural units of *passenger* and *locomotor*, which was studied by Perry (1992), acts as a natural shock absorber. Therefore, it is not surprising that the movement of this kind of structure should be characterized by a considerable degree of symmetry in the two joints. Given man’s desire to move during gait with equal steps, both our own research and data of, for example, Menard et al. (1992) revealed this symmetry in the sagittal plane. Also data of Smith et al. (2002) confirmed these findings. However, the authors found evidence of slight differences in the pattern of hip movement in other (frontal and transverse) planes.

Based on the analysis of the literature, our own observations, practices, and the results of the present study, we can tentatively posit that the higher (in terms of anatomy) the joint of the lower limb is located, the more similar (in terms of quality) is its profile of movement on both sides of the body.

### Recommendation for the future

The overall aim of this research was to develop a new approach identifying asymmetry in the able-bodied gait in terms of differences between the joints of lower limbs in women and men. However, additional investigation is necessary to provide a more comprehensive analysis of gait patterns, which also considers asymmetry in dynamic aspects of such movement. Another focus of the further research would be comparison of the existing symmetry indices to verify which one is the most appropriate in normal ambulation.

## Conclusions

The obtained results concerning the asymmetry of gait allowed us to formulate the following conclusions:

1. The values of spatio-temporal gait parameters measured bilaterally did not differ significantly in healthy young men and women.
2. Asymmetry in the pattern of motion of both lower extremity joints was revealed in angular changes in the sagittal plane.
3. The highest values of the relative asymmetry index, which describes the changes of the joint angles, was noted for the ankle; RAI reached the lowest level in the knee and hip joints.
4. An evaluation of symmetry must consider a wide spectrum of parameters since the conclusions following from an incomprehensive analysis may be conflicting.
5. Despite moving at similar gait speed, there are differences in asymmetry between men and women in terms of spatio-temporal and angular variables.

## Figures and Tables

**Table 1:** Mean values of the selected morphological parameters in women and in men (mean±standard deviation): BMI – Body Mass Index (ratio of body mass [in kilograms] per body height [in meters squared])

Sex	Body height [m]	Body mass [kg]	BMI [kg/m^2^]
**Women**	1,66±0,05	58,23±5,56	21,13±1,27
**Men**	1,82±0,07	78,14±10,16	23,59±1,65

The BMI classifications used in this study were based on the World Health Organization’s definitions of normal weight (BMI 18.5 to 24,9 kg/m^2^), overweight (BMI 25 to 29,9 kg/m^2^).

**Table 2:** Basic kinematic parameters of gait at natural preferred-speed in women and men

	**Women**			**Men**		
	*x̄*±SD	min	max	*x̄* ±SD	min	max
**Velocity** [m/s]	1,56±0,09	1,41	1,69	1,54±0,08	1,43	1,69
**Frequency** [steps/min]	123±5,64	109	134	118±5,2	112	126

**Table 3:** Basic values of kinematic parameters recorded during gait in women (W) and in men (M) (**t**_GC_ and **s** – respectively: the time and the stride, SS and DS – respectively: single and double support time, R - right, L - left limb)

	t_GC_* [s]					
	**R**			**L**		
	*x̄* ±SD	min	max	*x̄* ±SD	min	max
**W**	0,98±0,04	0,90	1,09	0,98±0,04	0,90	1,10
**M**	1,02±0,04	0,95	1,07	1,02±0,04	0,95	1,09
	s* [m]					
	**R**			**L**		
	*x̄* ±SD	min	max	*x̄* ±SD	min	max
**W**	1,52±0,07	1,39	1,68	1,52±0,07	1,39	1,67
**M**	1,57±0,1	1,42	1,74	1,57±0,1	1,44	1,73
	SS** [s]					
	**R**			**L**		
	*x̄* ±SD	min	max	*x̄* ±SD	min	max
**W**	0,38±0,02	0,36	0,43	0,39±0,02	0,37	0,44
**M**	0,40±0,02	0,37	0,45	0,40±0,02	0,37	0,44
	**DS [s]**					
	**R**			L*		
	*x̄* ±SD	min	max	*x̄* ±SD	min	max
**W**	0,21±0,02	0,17	0,25	0,21±0,02	0,17	0,25
**M**	0,21±0,04	0,18	0,26	0,22±0,02	0,18	0,26

Statistical significance of the differences between W and M:

* p<0,05,

** p<0,01.
